# Modes of transmission of SARS-CoV-2 and evidence for preventive behavioral interventions

**DOI:** 10.1186/s12879-021-06222-4

**Published:** 2021-05-28

**Authors:** Lucas Zhou, Samuel K. Ayeh, Vignesh Chidambaram, Petros C. Karakousis

**Affiliations:** 1 Department of Medicine, Johns Hopkins University School of Medicine, Baltimore, MD USA; 2 Department of International Health, Johns Hopkins Bloomberg School of Public Health, Koch Cancer Research Building, 1550 Orleans St., Room 110, Baltimore, MD 21287 USA

## Abstract

COVID-19 is a novel disease caused by SARS-CoV-2. During the global vaccination rollout, it is vital to thoroughly understand the modes of transmission of the virus in order to prevent further spread of variants and ultimately to end the pandemic. The current literature suggests that SARS-CoV-2 is transmitted among the human population primarily through respiratory droplets and, to a lesser extent, via aerosols. Transmission appears to be affected by temperature, humidity, precipitation, air currents, pH, and radiation in the ambient environment. Finally, the use of masks or facial coverings, social distancing, and hand washing are effective public health strategies in reducing the risk of exposure and transmission. Additional research is needed to further characterize the relative benefits of specific nonpharmaceutical interventions.

## Introduction

COVID-19 is caused by a beta-coronavirus named SARS-CoV-2 due to its genetic similarities with SARS-CoV-1 (the etiological agent of Severe Acute Respiratory Syndrome (SARS)) [1]. The first cases of COVID-19 were reported in China in late December 2019, and currently, cases have been reported in most countries around the world with very significant morbidity and mortality [2, 3]. The disease, which in 1 year caused over 120 million infections and 2.5 million deaths worldwide, was declared a pandemic by the World Health Organization (WHO) in March 2020 [4].

Identifying and recognizing the relative importance of the factors promoting SARS-CoV-2 transmission is critical to implementing public health strategies to contain the pandemic and future outbreaks of related beta-coronaviruses. This review summarizes the available information on known modes of human-to-human transmission of SARS-CoV-2, environmental factors that affect transmission of the virus, and evidence supporting the use of specific nonpharmaceutical and public health interventions to limit its spread.

## Modes of transmission

### Respiratory transmission: respiratory droplets and aerosols

Prior studies on SARS-CoV-1 suggest that respiratory droplets are the primary mode of transmission for beta-coronaviruses [5]. The transmission of SARS-CoV-2 occurs primarily through respiratory droplets and aerosols generated during coughing or sneezing, which may land on the nose, mouth or eyes [6]. Aerosols and droplets are considered different parts of a continuum, and various proportions are emitted from the individual depending on the type of activity performed, such as talking, coughing or sneezing [7]. Although large respiratory droplets (> 5 μm) tend to fall out of the air rather quickly, aerosols containing smaller particles (< 5 μm) can travel across greater distances [8, 9]. The possibility of SARS-Cov-2 transmission via aerosols was underscored by a provocative study by van Doremalen et al., who reported that SARS-CoV-2 can remain viable in aerosols for up to 3 h, with less than 20% reduction in infectious titer during this time period [10]. It is important to note, however, that this study utilized a nebulizing machine to generate very small (< 5 μm) airborne particles containing SARS-CoV-2 (10^5.25^ 50% tissue-culture infectious dose [TCID_50_] per milliliter), and it is unclear if typical patients with COVID-19 generate similar aerosols containing this burden of viable virions [11].

Shen et al. investigated a COVID-19 outbreak during an outdoor religious ceremony in China [12]. Among the 31 people infected, 24 of them travelled to the ceremony in the same bus as the index case, and the remaining 7 had close contact with the index case at the ceremony. None of the patients who travelled in a different bus developed the infection. Case density was non-significantly higher in the area closest to the index case, who was pre-symptomatic. These findings suggest that airborne, and possibly aerosol, transmission of SARS-CoV-2, likely due to air recirculation and poor ventilation within the bus, were associated with the higher attack rate [12].

In an analysis of the Skagit valley choir outbreak, Hamner et al. concluded that respiratory droplets were responsible for transmission. During a rehearsal, 52 out of the 60 naïve attendees (86.7%) were infected. Although seating charts were not provided, because the choir sat in a grid of approximately 4 ft deep by 13 ft wide, and the range of expelled respiratory droplets is approximately 6 ft, the index case could have infected the entire choir if he/she were positioned in the middle of the seating grid [13]. Another analysis of the same outbreak concluded that, because precautions that limited the potential for direct contact and droplet-facilitated infection were employed, such as the use of hand sanitizer and the avoidance of handshakes and hugging, the choir members likely become infected with SARS-CoV-2 by inhaling the aerosols from the index case, since singing can generate large amounts of aerosols [14].

After investigating the potential contributions of respiratory droplets, aerosols, and fomites among new cases of COVID-19 aboard the Diamond Princess cruise ship, Azimi et al. concluded that short-range aerosols were the dominant mode of transmission [15]_._ Investigating the same outbreak, Xu et al. did not find a substantial increase in cases after the stay-in-room policy was implemented, leading them to conclude that the virus was not spread through the central air-conditioning to individual rooms, and that long-range aerosol transmission may not be possible [16]. These data are compatible with respiratory droplet or short-range aerosol transmission.

In a case study of a spreader event in a restaurant in China, more infections were reported among members of the family seated downstream of the index case, along the flow of the air conditioner, than in the family seated upstream [17]. Santarpia et al. found that 63.2% of air samples from 11 residential isolation rooms in the Nebraska Biocontainment Unit and National Quarantine Unit and 58.3% of samples taken outside the rooms in the hallways tested positive for SARS-CoV-2 by RT-PCR [18]. The air samples taken near infected patients had a higher concentration of RNA than those taken farther than 2 m away, near the door of the room (4.07 vs 2.48 copies/L of air, respectively) [18]. Furthermore, viral RNA was detected in all the samples from the floor under beds. This could be explained by airflow modeling, which predicted that some fraction of the airflow was directed under the patients’ beds [18]. Taken together, these results suggest that SARS-CoV-2 is transmitted through the airborne route, primarily through respiratory droplets and short-range aerosols [18].

It should be noted that conclusions may be limited from SARS-CoV-2 environmental sampling studies reporting only RT-PCR data. This is because these studies reflect the presence of viral RNA rather than viable, transmissible virus. Nevertheless, additional research is needed to assess the contributions of short-range aerosols vs. respiratory droplets in the transmission of SARS-CoV-2.

### Fomite transmission

Early in the pandemic, direct contact with fomites harboring SARS-CoV-2 and subsequent contact with the respiratory or ocular mucosa was implicated in virus transmission. In support of this possibility, infectious SARS-CoV-1 particles (P9 strain) were found to persist for up to 4–5 days on metal, paper surfaces, wood and plastic surfaces [2]. A study of inanimate surfaces in two hospitals in Bangkok and Taipei detected SARS-CoV-1 RNA by RT-PCR in 12 of 43 swabs from patient rooms and 10 of 47 swabs from other parts of the hospital. However, all cultures of these samples showed no growth [19]. The surface stability of SARS-CoV-2 appears to differ slightly from that of SARS-CoV-1, as the former remains viable on copper metal for up to 4 h, on cardboard for up to 1 day, and on plastic and stainless steel for 2–3 days [10, 11]. However, it is important to note that the inoculum used to infect the surfaces in the latter study was significantly higher than that shed by infected persons [10, 11].

In a study by Ong et al.*,* samples taken from bathroom surfaces, including toilets and sinks, in a clinical center treating patients with COVID-19 yielded positive results for viral RNA, suggesting that these surfaces could represent conduits for virus transmission [20]. A similar study was conducted by Guo et al. at Huoshenshen Hospital in Wuhan. SARS-CoV-2 RNA was detected in samples collected from the floors of the intensive care unit (70%), general wards (15.4%), and the hospital pharmacy (100%) [8]. Interestingly, no patients entered the pharmacy before sample collection, suggesting that SARS-CoV-2 might be carried on the soles of the shoes of medical staff and subsequently aerosolized during walking. In particular, smaller particles (< 2.5 μm) could be catapulted from personal protective equipment during removal, while the larger ones (> 2.5 μm) might be stirred from the floor by the shoes of the medical staff [21]. The virus was also detected by PCR on other surfaces, like computer mice, trash cans, sickbed handrails, and doorknobs [8]. Liu et al. showed that samples collected from hospital areas accessible only to medical staff yielded greater amounts of SARS-CoV-2 RNA than those collected from patient-accessible regions [21].

Although these data suggest that fomites might contribute to community and nosocomial spread of SARS-CoV-2, one should interpret the findings with caution. In studies investigating the viability of the virus on inanimate surfaces, very high inocula were used, and other environmental sampling studies tested for viral RNA rather than viable virus [22]. Additional studies, with conditions more reflective of typical and real-life SARS-CoV-2 exposure conditions, should be conducted [22, 23].

### Other body fluids

Studies have shown the presence of SARS-CoV-2 in human saliva. In a study by To et al.*,* among 12 confirmed cases of COVID-19, 11 had virus detectable by PCR, and three patients had viable virus in their saliva [24]. These findings could indicate direct infection of the salivary glands by SARS-CoV-2, or the transit of respiratory secretions from the nasopharynx or lower airways through the mouth. This could allow for the spread of SARS-CoV-2 through the exchange of saliva, e.g., by kissing or sharing toothbrushes [25].

Ocular involvement has not been described with MERS-CoV and SARS-CoV [26]. On the other hand, in a study by Loon et al., the tear samples of three patients with SARS were shown to have SARS-CoV-1 RNA [27]. Xia et al. reported the presence of SARS-CoV-2 RNA in the tears of one of 30 COVID-19 patients with conjunctivitis [28]. However, viable virus could not be cultured from the samples in either of the above studies.

To study the potential transmission of SARS-CoV-2 through feces, Zhang et al. collected anal and oral swabs from 16 patients with COVID-19, and found detectable RNA in anal swabs from 4 and 6 patients 0 and 5 days since diagnosis, respectively [29]. Conversely, oral swabs were positive by RT-PCR in 8 and 4 patients on day 0 and day 5, respectively. It is important to note that virus viability in fecal samples was not tested in this study [29]. Nonetheless, it has been suggested that feces could be deposited on bathroom fomites as patients attempt to clean themselves, and these could then be deposited onto respiratory mucosal surfaces [30]. Ding and colleagues detected a robust viral RNA signal in 4 out of the 107 samples collected from surfaces in the bathroom area in a COVID-19 dedicated hospital [31]. It is also possible that aerosolization of SARS-CoV-2 from feces during toilet flushes may result in transmission [31].

Qiu et al. were unable to detect virus by RT-PCR in vaginal samples collected from 10 women with COVID-19 [32]. As only one sample was collected from each woman, and some of them were collected more than 17 days after infection, the window for detection of SARS-CoV-2 could have been missed. Furthermore, since all of them were postmenopausal, it is unclear if these findings are generalizable to younger women [32].

Pan et al. analyzed 6 male patients with symptoms suggestive of viral orchitis [33], but were unable to detect SARS-CoV-2 RNA in their semen. Similarly, Song et al. found that semen samples collected from each of 12 patients recovering from COVID-19 and the post-mortem testes samples from a deceased individual tested negative for SARS-CoV-2 RNA [34]. In contrast, Li et al. recovered SARS-CoV-2 RNA from the semen of 6 of 38 patients with active COVID-19 [35]. Also, it was noted that three of the six patients with RNA-positive semen died subsequently, but all 32 semen-negative patients survived. This suggests that the presence of SARS-CoV-2 in semen might be a marker of severe disease. Although reproductive fluids are unlikely to represent a significant mode for transmission of SARS-CoV-2, additional studies are needed to fully assess the risk patients with COVID-19 pose to their sexual partners.

## Environmental factors

### Temperature, humidity, and precipitation

Kampf et al. noted that high temperatures (above 30 °C) reduce the viability of coronaviruses, while low temperatures (4 °C) prolong their persistence to over 28 days [36]. Darnell et al. found that progressively higher temperatures resulted in accelerated inactivation of SARS-CoV-1 [37], leading the authors to conclude that pasteurization may be highly effective in inactivating the virus [37]. Humidity may also affect coronavirus survival in the environment. HCoV-299E had improved viability at 50% humidity compared to 30% [36]. Chan et al. found that subjecting SARS-CoV-1 to > 95% humidity produced a smaller reduction in viral titer than at 40–50% humidity [38]. Sobral et al. estimated that for every one-degree Fahrenheit increase in ambient temperature, the number of confirmed COVID-19 infections decreased by 1.44 cases/day, while for each inch increase in precipitation/day, there was an increase of 56.01 cases/day [39]. These results suggest that the persistence of SARS-CoV-2 might correlate negatively with temperature and positively with humidity.

However, other studies have suggested that the relationship between relative humidity and coronavirus stability in the environment may not be linear. Casanova et al. observed that SARS-CoV-1 survival was greatest at 20 and 80% humidity, and lowest at 50% [40]. In addition to low average temperature and mild diurnal temperature range, Liu et al. concluded that low humidity favors COVID-19 transmission [41]. Eykelbosh reported that high relative humidity reduces airborne material and the viability of virus in airborne particles and on surfaces, thereby decreasing the risk of COVID-19 transmission in humid environments [42].

Using transmission data of two beta-coronaviruses, HCoV-HKU1 and HCoV-OC43, Baker et al. generated computational models to simulate the global spread of SARS-CoV-2. The results from the first model showed that the basic reproduction number (R_0_) might decline or remain the same as the humidity increases [43]. This suggests that SARS-CoV-2 survival may be inversely correlated with humidity. The second model suggests that pandemic size is dependent on latitude: in the northern hemisphere, the differences in simulations performed in New York, London, and Delhi were not significant, despite all three having very different climates. All tropical locations had longer duration and lower intensity pandemics than northern locations. The third model concluded that if the R_0_ is higher, control measures are more important in reducing the impact of the pandemic. If R_0_ is lower, climate plays a more substantial role in determining the magnitude of the pandemic [43].

The majority of the evidence suggests that elevated temperature adversely affects SARS-CoV-2 viability, however the precise effect of humidity remains unclear. More research is needed to elucidate the dependence of SARS-CoV-2 transmission on climate, especially at lower temperatures and humidity.

### Radiation

UV light, with a wavelength of 254 nm, is an effective biocide. However, excessive exposure can cause eye and skin irritation in the short-term, and scarring in the long term [44, 45]. In contrast, UVC light, with a wavelength of 207–222 nm, is believed to be safer [46]. Buonanno et al. noted that when aerosolized HCoV-OC43 was exposed to UVC levels well below the regulatory exposure limit of 3 mJ/cm^2^, 99.9% of particles were inactivated. Thus, it was estimated that 25 min of exposure to 3 mJ/cm^2^ of UVC light could inactivate 99.9% of SARS-CoV-2 particles with minimal risk of harm to nearby humans [46].

Darnell et al. found that gamma radiation from Cobalt-60 and UVA had no effect on SARS-CoV-1 inactivation, even after 15 min of exposure [37]. However, UVC light emitted from a source 3 cm from the sample at an intensity of 4016 μW/cm^2^ partially inactivated SARs-CoV-1 after 1 min of exposure and completely inactivated [≤1.0 TCID_50_ (log_10_)] the virus after 15 min*.* Duan et al. achieved inactivation of SARS-CoV-1 after 60 min by using a light intensity > 90 μW/cm^2^ at a distance of 80 cm [2]. Because the CDC has used a much higher dose of 2 × 10^6^ rad of gamma radiation from Cobalt-60 to inactivate potential SARS-CoV-infected serum specimens for study in BSL2 laboratories compared to the 1.5 × 10^4^ rad used by Darnell et al., these results do not invalidate the CDC’s radiation sterilization guidelines [37].

It is important to note these virus inactivation approaches may not be feasible in practice. Mills et al. found that a 1 J/cm^2^ dose of UVC radiation on circular coupons prepared from N95 respirators was sufficient to kill MERS-CoV and SARS-CoV-1 [47]. However, such a dose was insufficient to sterilize a sizable number of soiled facepieces and straps [47]. Narla et al. concluded that it is essential to make sure that no shadowing materials, e.g., cosmetics or sunscreen, are present on PPE when sanitizing to ensure thorough cleaning [48]. Further research is needed to determine the necessary radiation dosages to achieve virus sterilization.

### pH

Extremely basic and acidic conditions significantly reduce SARS-CoV-1 viability [37]. Exposure of SARS-CoV-1 to pH 12–14 for 1 hour and pH of 1–3 at 25–35^°^C completely inactivated the virus. In contrast, a pH range of 5–9 was generally hospitable to SARS-CoV-1, regardless of temperature. Further studies are needed to test the translatability of these results to SARS-CoV-2 [37].

## Measures to prevent transmission

Doung-Ngern et al. conducted a case-control study in Thailand to determine the extent to which preventive measures independently reduce COVID-19 transmission [49]. They found that maintaining an interpersonal distance of greater than 1 m reduced the odds ratio of developing COVID-19 to less than 0.2. The next greatest reduction was that produced by always wearing any face mask (nonmedical or medical mask), followed by occasional use of a mask, as each practice reduced the odds ratio to just over 0.2. Finally, limiting interpersonal contact to less than 15 min and at least sporadic handwashing reduced the odds ratio to around 0.3 [49].

### Use of face masks or coverings

The WHO and CDC have recommended the routine universal use of face masks or coverings to prevent SARS-CoV-2 transmission during the pandemic [16, 50]. Since a significant drop in COVID-19 incidence in New York City and Italy coincided with mandated use of face masks/coverings, Zhang et al. concluded that airborne transmission was the dominant route for the spread of COVID-19 [51]. However, Leung et al. reported that facemasks or coverings may also prevent the spread of SARS-CoV-2 via respiratory droplets [52].

In a study at the Boston’s Mass General Brigham hospitals, the SARS-CoV-2 positivity rate changed from a daily increase of 1.16% per day to a daily decrease of 0.49% per day after instituting a mandatory masking policy for all healthcare workers and patients [53]. Similarly, Lyu et al. studied the association between masking mandates and transmission rates across the U.S. and found that daily case rates declined by 0.9, 1.1, 1.4, 1.7, and 2% within 1–5, 6–10, 11–15, 16–20, and 21 or more days, respectively, after masking mandates [54]. All case rate declines were statistically significant, indicating that masks are effective in reducing SARS-CoV-2 transmission. A case-control study by Fisher et al. found that visiting restaurants and coffee shops that offer dine-in options, where masks are removed while eating and drinking, were associated with higher SARS-CoV-2 positivity [55].

N95 respirators can filter 95% or more of particles as small as 0.3-μm in diameter [56]. Leung et al. found that even surgical masks are capable of filtering both SARS-CoV-2-containing respiratory droplets and aerosols in exhaled breath [52]. However, an earlier study found that surgical masks reduced the emission of respiratory droplets, but not of aerosols containing influenza virus [57]. The reason for the differences in efficacy of surgical masks in preventing expulsion of SARS-CoV-2 and influenza by the wearer remains unexplored.

Furthermore, Verma et al. found that folded handkerchiefs and homemade facemasks reduced the size of the particle cloud, but noted that particles leaked around the edges and through the material of such masks [58]. In a study by Chan et al. assessing the efficacy of surgical masks to prevent COVID-19 infection in golden hamsters, they found that orienting the mask such that the fluid-repellent blue layer faced the naïve hamsters resulted in fewer infections than if the mask was reversed [59].

A contact tracing investigation of a mechanically ventilated patient with delayed COVID-19 diagnosis at a Singapore hospital identified 41 health care workers who had exposure to aerosol-generating procedures for at least 10 min at a distance of less than 2 m from the patient [60]. Of these contacts, 85% wore surgical masks during the aerosol-generating procedures, while the remainder wore N95 masks. None of the 41 health care workers acquired COVID-19 infection, leading the authors to conclude that surgical masks, hand hygiene, and other standard procedures protected them from becoming infected [60]. These findings are consistent with prior studies showing that N95 masks are not significantly superior to surgical masks for preventing influenza infection in health care workers [61]. These findings offer some reassurance that surgical masks may be acceptable alternatives in high-risk settings where N95 masks are not available.

As in the nosocomial setting, the use of face masks also appears to be effective in preventing SARS-CoV-2 transmission in the community. In a hair salon in Missouri, two hair stylists attended to 139 clients between first symptom onset and testing positive. One stylist wore a double-layered cotton face covering, and the other stylist wore either a double-layered cotton face covering or a surgical mask at work during the time period before and while feeling ill. All the clients at the salon wore face masks during the entire appointment. None of the 139 clients reported developing COVID-19, and none of the 67 clients who underwent nasopharyngeal swab for RT-PCR testing for SARS-CoV-2 had positive results. However, four housemates of stylist A subsequently developed COVID-19 infection [62, 63]. These findings show that face coverings provide effective protection against virus transmission, including in indoor airspaces, and also that the home environment, especially when masks are not routinely used, represents one of the most common venues of transmission [64]. A trial by Bundgaard et al. showed that mask wearing did not reduce the risk of SARS-CoV-2 infection in the wearer. However, there were a number of limitations to this study, including poor adherence to mask wearing in the intervention group [65]. Also, this study did not investigate the potential protection against COVID-19 offered to the greater community due to mask wearing [65, 66].

Since face masks or coverings significantly reduce the expulsion of respiratory droplets and aerosols containing SARS-CoV-2 by infected individuals [52], their universal use could reduce community transmission during the pre-symptomatic stage or in asymptomatic individuals. This is particularly important since various studies suggest that 40–80% of COVID-19 cases are asymptomatic [67, 68], although such individuals may still be contagious [69, 70]. Also, He et al., found that patients with COVID-19 infection begin shedding viral particles up to 3 days prior to displaying symptoms, with peak infectiousness occurring up to 2 days before symptom onset [71], leading the authors to conclude that 25–69% of COVID-19 transmission occurs during the pre-symptomatic period [71]. Several recent meta-analyses also support the conclusion that face mask use significantly reduces COVID-19 transmission and infection rates [72, 73].

Many U.S. health care systems have implemented the use of eye protection in addition to face masks/covering during interactions of medical personnel and staff with low-risk inpatients and ambulatory patients [74]. Huang et al. reported the case of a man potentially infected with SARS-CoV-2 through the ocular membranes during an inspection in Wuhan [75]. Despite using an N95 mask, he developed redness of the eyes several days before the onset of pneumonia. A study by Chu et al. found that the adjusted odds ratio for infection in people using eye protection versus those who did not was 0.12 to 0.39, which lends further support for the use of eye protection [76]. More studies and data are needed before recommending the routine use of face shields or safety goggles in public spaces.

### Social distancing

Although the maximum transmission distance of aerosolized SARS-CoV-2 is not definitively known, the CDC suggests that carriers can infect people within a 6-ft radius [13]. This is supported by the systematic review by Chu et al., which found that virus transmission was significantly lower with physical distancing of 1 m or more, compared with a distance of less than 1 m (pooled adjusted odds ratio of 0.18 [95% CI 0·09 to 0·38]) [52]. These results are consistent with respiratory droplets, and perhaps short-range aerosols, as the primary mode by which SARS-CoV-2 is transmitted. Nevertheless, longer range aerosol transmission of SARS-CoV-2 is still possible. Some researchers believe that aerosolized SARS-CoV-2 particles can be dispersed to distances up to 10 ft [77], while Guo et al. suggested that the maximum transmission distance of aerosolized SARS-CoV-2 could be 4 m [8]. A recent study compared the incident cases of SARS-CoV-2 among students and staff in Massachusetts public schools by comparing districts with different physical distancing requirements [78]. This study showed that among 251 eligible school districts, the case rates among students and staff were similar in the districts with ≥3 ft versus ≥6 ft of physical distancing requirement [78]. More research is needed to conclusively determine the minimum separation required for social distancing to be effective.

Gallaway et al. observed that shortly after the lifting of stay-at-home orders, case counts increased by 151% in the first 2 weeks of June in Arizona. Following the implementation of mask-wearing mandates and social distancing, case counts fell 75% over 4 weeks [79]. Because pre-symptomatic and symptomatic transmission comprise the majority of the total R_0_ of SARS-CoV-2, Ferretti et al. concluded that preventing interactions between infected individuals and their contacts is the best way to halt the COVID-19 epidemic [80].

However, emerging evidence suggests that sharing indoor space is a major SARS-CoV-2 infection risk. Qian et al. investigated the circumstances surrounding 7324 cases of COVID-19, of which only two occurred outdoors [64]. These data suggest that while self-isolation and quarantine may help to reduce the overall transmission rate of COVID-19, it might increase the risk of indoor transmission [64]. Kupferschmidt also suggested that home isolation together with other individuals might not be ideal to prevent COVID-19 transmission [81]. In an epidemiological investigation of an outbreak at an indoor call center in South Korea, 97 of 1143 people tested positive, with an attack rate of 43.5% (95%CI, 36.9–50.4) [82]. SARS-CoV-2 is thought to have a dispersion factor ‘k’ slightly higher than that of MERS, another related coronavirus. Thus, being in close proximity in enclosed spaces is an important factor contributing to SARS-CoV-2 transmission [81].

### Hand hygiene

The WHO and CDC recommend frequent handwashing and hand sanitization using at least 70% alcohol solutions to prevent spreading and contracting COVID-19 [83–85]. These recommendations were corroborated by Kratzel et al., who found that both 80% ethanol and 75% 2-propanol reduced the amount of viable SARS-CoV particles by over 99.9% [86]. Kampf et al. observed that ethanol was more effective than 2-propranol against SARS-CoV, with a 10^5.5^ reduction in viral infectivity in 30 s by 95% ethanol, and 10^4.0^ reduction with 75% 2-propranol [36]. Darnell et al. reported that neither 1:4000 dilution formalin nor glutaraldehyde could completely inactivate SARS-CoV-1 at 4^o^ C. However, at higher temperature, formalin and glutaraldehyde inactivated SARS-CoV-1 particles in two to 3 days [36, 37]. More research is needed to determine the most effective biocide against SARS-CoV-2.

In the case-control study by Doung-Ngern et al., handwashing was associated with a reduced odds ratio of transmission (0.34; 95%CI 0.13–0.87), but the protection is significantly lower when compared to mask wearing [49]. Given that hand hygiene is a relatively straightforward and benign public health measure, it seems prudent to continue to recommend its implementation. However, given the low likelihood of fomites as vectors of transmission relative to respiratory droplets and short-range aerosols, the emphasis of public health messaging should be placed on mask wearing and physical distancing for preventing SARS-CoV-2 transmission.

## Maintaining preventive behavioral interventions during the COVID vaccine rollout

A number of COVID-19 vaccines are currently being administered. While they have the potential to curb the pandemic, it is important to note that until the threshold for herd immunity is met, it remains important to implement behavioral interventions to minimize transmission through various routes discussed above [87]. Such interventions will also prevent the evolution of novel variants against which current vaccines might be less effective [88]. Though it is not clear what this threshold is, computational models predict that even in the most ideal of circumstances, vaccines need to be administered to a majority of the population in order to significantly reduce SARS-CoV-2 transmission. Mukandavir et al. suggests that in South Africa, even with a 100% effective vaccine, over 65% of the population must be vaccinated for the COVID-19 outbreak to be contained [89]. More realistically, a vaccine with 70% efficacy must be administered to almost 95% of the South African population to contain the epidemic [89]. A model of the United States predicts that if face mask use stops completely, between 33 and 58% of the population would need to be vaccinated with a 100% effective vaccine to suppress the epidemic [90]. This threshold is roughly the same in the realistic scenario where face mask use is reduced by 50% and vaccine effectiveness is 80% [90]. As more research is done to determine the threshold for herd immunity, the sustained implementation of preventive behavioral interventions remains important in preventing ongoing community transmission of SARS-CoV-2.

## Conclusion

Current knowledge suggests that the transmission dynamics of SARS-CoV-2 is similar to that of other beta-coronaviruses, with the respiratory system being the most common point of entry and respiratory droplets and aerosols being the most common modes of transmission. Based on epidemiological and modeling data, the CDC and WHO have developed guidance to prevent transmission of the infection. There is substantial evidence to show that the regular use of any face mask, and social distancing can effectively reduce the transmission of this virus. There is not enough evidence on the type of mask that is best for the prevention of transmission of infection in the community. Handwashing with soap or the use of alcohol sanitizer have also been shown to reduce the risk of infection in the community to a certain extent. More research is needed on the sterilization of instruments and surfaces, and the importance of the role of fomites in the transmission of the infection. Further research on SARS-CoV-2 will also expand our understanding of the transmission of other respiratory viruses.

## Data Availability

As this is a review article, primary data were not obtained.
